# Therapeutic efficacy of an Ad26/MVA vaccine with SIV gp140 protein and vesatolimod in ART-suppressed rhesus macaques

**DOI:** 10.1038/s41541-022-00477-x

**Published:** 2022-05-18

**Authors:** John D. Ventura, Joseph P. Nkolola, Abishek Chandrashekar, Erica N. Borducchi, Jinyan Liu, Noe B. Mercado, David L. Hope, Victoria M. Giffin, Katherine McMahan, Romas Geleziunas, Jeffrey P. Murry, Yunling Yang, Mark G. Lewis, Maria G. Pau, Frank Wegmann, Hanneke Schuitemaker, Emily J. Fray, Mithra R. Kumar, Janet D. Siliciano, Robert F. Siliciano, Merlin L. Robb, Nelson L. Michael, Dan H. Barouch

**Affiliations:** 1 Center for Virology and Vaccine Research, Beth Israel Deaconess Medical Center, Harvard Medical School, Boston, MA 02215 USA; 2 Gilead Sciences, Foster City, CA 94404 USA; 3 Bioqual, Rockville, MD 20852 USA; 4 Janssen Infectious Diseases and Vaccines, 2301 Leiden, The Netherlands; 5 Department of Medicine, Johns Hopkins University School of Medicine, Baltimore, MD USA; 6 US Military HIV Research Program, Walter Reed Army Institute of Research, Silver Spring, MD 20910 USA; 7 Ragon Institute of MGH, MIT, and Harvard, Cambridge, MA 02139 USA

**Keywords:** Vaccines, Vaccines

## Abstract

Developing an intervention that results in virologic control following discontinuation of antiretroviral therapy (ART) is a major objective of HIV-1 cure research. In this study, we investigated the therapeutic efficacy of a vaccine consisting of adenovirus serotype 26 (Ad26) and modified vaccinia Ankara (MVA) with or without an SIV Envelope (Env) gp140 protein with alum adjuvant in combination with the TLR7 agonist vesatolimod (GS-9620) in 36 ART-suppressed, SIVmac251-infected rhesus macaques. Ad26/MVA therapeutic vaccination led to robust humoral and cellular immune responses, and the Env protein boost increased antibody responses. Following discontinuation of ART, virologic control was observed in 5/12 animals in each vaccine group, compared with 0/12 animals in the sham control group. These data demonstrate therapeutic efficacy of Ad26/MVA vaccination with vesatolimod but no clear additional benefit of adding an Env protein boost. SIV-specific cellular immune responses correlated with virologic control. Our findings show partial efficacy of therapeutic vaccination following ART discontinuation in SIV-infected rhesus macaques.

## Introduction

Although antiretroviral therapy (ART) is highly effective, there is currently no cure for HIV infection, largely due to the persistence of a latent viral reservoir in resting memory CD4+ T cells^1–3^. Current efforts to cure HIV infection involve the identification, activation, and eradication of the latent reservoir through immunologic interventions^4–6^. One such strategy includes latency reversing agents (LRAs) to activate latent viral reservoirs together with an immunologic method to identify and eliminate reactivated cells, a strategy often referred to as “shock and kill”^4,5,7–11^.

Toll-like receptor 7 (TLR7) is an endosomal innate pattern-recognition receptor (PRR) that recognizes polyuridine tracts commonly found in the genomes of single-stranded RNA viruses^12,13^. TLR7 is primarily expressed by plasmacytoid dendritic cells and B cells^12,14,15^. Engagement of TLR7 leads to cellular activation and the production of both proinflammatory cytokines and Type I interferon via two intracellular signaling axes involving the transcription factors NF-κB and interferon regulatory factor 7, respectively^12^. TLR7 signaling results in indirect activation of CD4+ T cells, which may account for activation of HIV in vitro^16^, and TLR7 agonists are being explored in viral eradication strategies as LRAs and activators of innate immune responses^17^.

TLR7 agonists have previously been used in combination with both therapeutic SIV vaccination as well as with broadly neutralizing antibodies (bNAb) in SIV and SHIV infected rhesus macaques while on ART to reduce viral reservoirs and confer virologic control after ART discontinuation. Combined delivery of the TLR7 agonist GS-986 and therapeutic vaccination regimen consisting of Ad26 and MVA vectors expressing SIV_smE543_ *gag–pol–env* led to post-ART virologic control in 33% of animals^18^. Vesatolimod (GS-9620) is an oral TLR7 agonist and close analog of GS-986 that similarly induces TLR7 signaling^19–21^. In a recent phase 1b clinical trial, HIV-1 controllers exhibited a delay in viral rebound after receiving repeated doses of vesatolimod while on suppressive ART^22^. Furthermore, vesatolimod administration in combination with passive transfer of the HIV-1 V3-glycan-dependent bNAb PGT121 in ART-suppressed SHIV-infected rhesus macaques resulted in delayed viral rebound and a reduced viral reservoir in lymph nodes, leading to long-term virologic control in 5 of 11 treated animals^23^. These data suggested a strong connection between PGT121 antibody activity and viral reservoir burden and virologic control. In this study, we asked whether the addition of a SIV Envelope (Env) gp140 boost following Ad26/MVA vaccination would improve therapeutic efficacy in ART-suppressed, SIV-infected rhesus macaques.

## Results

### Study design

We investigated the immunogenicity and therapeutic efficacy of adding a SIV Env protein subunit boost with alum adjuvant to therapeutic Ad26/MVA vaccination in combination with vesatolimod administration in ART-suppressed, SIV-infected rhesus macaques. We intrarectally infected 36 outbred, Indian origin, rhesus macaques with 500 TCID_50_ SIV_mac251_. All animals were infected and initiated ART on day 7 following infection. ART consisted of daily subcutaneous administration of a preformulated cocktail of 5.1 mg/ml tenofovir disoproxil fumarate (TDF), 40 mg/ml emtricitabine (FTC), and 2.5 mg/ml dolutegravir (DTG) in a solvent containing 15% (v/v) kleptose at pH 4.2 at 1 ml/kg body weight, as we have previously described^18,23^. Animals were negative for protective MHC class I alleles Mamu-A*01, Mamu-B*08, and Mamu-B*17, and animals possessing both susceptible and resistant TRIM5α alleles were distributed evenly between all groups.

**Fig. 1:**
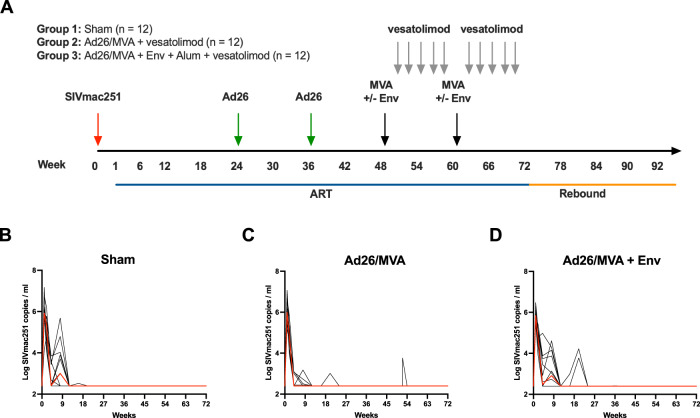
Ad26/MVA + Env protein subunit boost therapeutic vaccine strategy with vesatolimod treatment. **A** Therapeutic vaccine study design. **B**–**D** Log transformed plasma SIV_mac251_ viral loads (gag copies/ml plasma) from onset of infection to week 72 post-infection. Limit of detection (LOD) for the SIV viral load qRT-PCR assay used in the study was >250 *gag* copies/ml plasma. Red lines denote median values across all time points.

Animals were allocated into three experimental groups based on sex and age^1^: a sham control group (Sham, *n* = 12)^2^, a vaccine group comprised of two Ad26 vector primes and MVA vector boosts at weeks 24 and 36 and weeks 48 and 60 post-infection, respectively, each expressing SIV_smE543_ *gag–pol–env* immunogens (Ad26/MVA, *n* = 12), and^3^ an Ad26/MVA vaccine group with an SIV_smE543_ gp140 protein subunit boost with alum adjuvant with each MVA inoculation (Ad26/MVA + Env, *n* = 12) (Fig. 1A). The Ad26/MVA and Ad26/MVA + Env groups were both treated with five repeated doses of vesatolimod every 2 weeks after the first and second MVA ± Env boosts (Fig. 1A). After initial SIV_mac251_ infection, all animals exhibited high viral loads by day 7, followed by sustained virologic suppression after ART initiation (Fig. 1B–D). Several animals in each group required 8–24 weeks of ART for full virologic suppression, which was then generally maintained throughout the treatment period (Fig. 1B–D).

### Immunogenicity of Ad26/MVA therapeutic vaccination with TLR7 stimulation and an Env protein boost

**Fig. 2:**
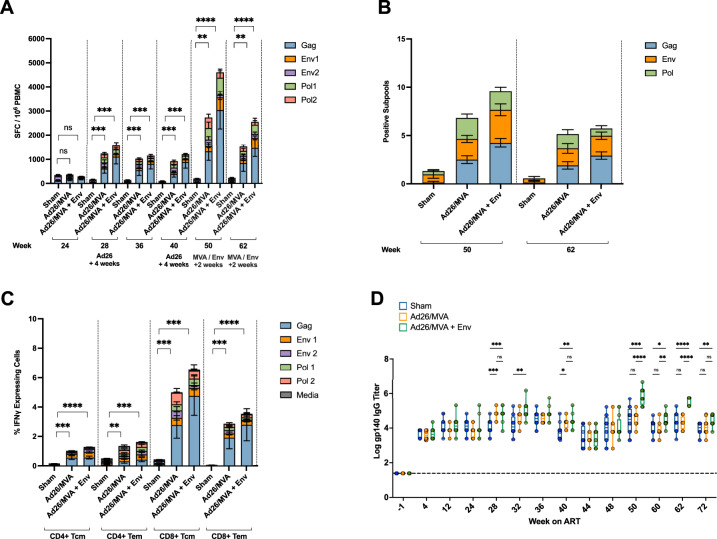
Vaccine-elicited cellular and humoral immune responses following therapeutic vaccination and vesatolimod treatment. **A** SIV-specific cellular immunity against Gag, Env, and Pol peptide subpools determined from IFNγ ELISpot assays performed on PBMCs sampled during Ad26/MVA and Ad26/MVA + Env therapeutic vaccination. Data shown as Spot Forming Cells (SFCs) per million cells. *P* values were derived from a Kruskal–Wallis test with Dunn’s correction for multiple comparisons of summed SFC counts for each treatment group. * < 0.05, ** < 0.01, *** < 0.001, **** < 0.0001. **B** Total immune breath of vaccine-induced SIV-specific IFNγ production quantified as the summation of total Gag, Env, and Pol subpools. **C** Flow cytometric assessment of CD4+ and CD8+ intracellular IFNγ secretion at week 62 post-infection following a 9 h stimulation with SIV_mac239_ Gag, Env, and Pol peptides. *P* values were derived from a Kruskal–Wallis test with Dunn’s correction for multiple comparisons from summed fractions of IFNγ positive cells for each treatment group. * < 0.05, ** < 0.01, *** < 0.001, **** < 0.0001. **D** SIV Env IgG titers measured by ELISA between 4 weeks prior to SIV_mac251_ infection (−4 weeks) and 72 weeks post-infection. Data shown as a box and whisker plot of log transformed values displaying the max and min values, median, and quartiles. *P* values were derived from a two-way ANOVA with Bonferroni correction for multiple comparisons. * < 0.05, ** < 0.01, *** < 0.001, **** < 0.0001.

Vaccine-elicited SIV-specific cellular immunity was assessed by IFN-γ ELISpot assays following SIV Gag, Env, and Pol peptide stimulation of peripheral blood mononuclear cells (PBMCs). Weeks 28 (4 weeks following the first Ad26 prime), 40 (4 weeks following the second Ad26 dose), 50 (2 weeks following the first MVA/Env subunit boost) and 62 (2 weeks following the second MVA/Env subunit boost) post-infection were selected for analysis. Both therapeutic vaccine regimens elicited robust SIV-specific T cell responses across the treatment period (Fig. 2A–C). Total cellular immune responses against Gag, Env, and Pol peptides increased substantially and were highly statistically significant when compared to sham controls following vaccination, with the highest responses detected 2 weeks following the first MVA/Env subunit boost at week 50 (Fig. 2A, *P* = 0.0026 and *P* < 0.0001 for Ad26/MVA and Ad26/MVA + Env groups compared to Sham, respectively, Kruskal–Wallis test with Dunn’s correction for multiple comparisons). Gag-specific responses constituted the highest frequency of total SIV-specific T cell responses at week 50 and trended higher in the Ad26/MVA + Env group, possibly as a result of the alum adjuvant (Fig. 2A; Supplementary Fig. 1A, B). Animals in the Ad26/MVA + Env group showed an expansion of Env-specific cellular responses at weeks 50, primarily against epitopes covered by the Env1 subpool (*P* = 0.0064 comparing Ad26/MVA and Ad26/MVA + Env groups, Mann–Whitney U test) and 62 (*P* = 0.0058) (Fig. 2A, Supplementary Fig. 1C–F). Animals in both the Ad26/MVA and the Ad26/MVA + Env groups also exhibited increased levels of cellular immune breadth as measured by positive responses against subpools of ten consecutive peptides (Fig. 2B). Taken together, Ad26/MVA therapeutic vaccination led to robust SIV-specific cellular immune responses with high magnitude and breadth of Gag, Env, and Pol -specific T cell responses.

Intracellular cytokine staining was also performed at week 62 post-infection to determine SIV-specific CD4+ and CD8+ T cell responses following vaccination (Fig. 2C, Supplementary Figs. 2, 3). CD4+ and CD8+ central and effector memory responses were diverse, covering epitopes across SIV Gag, Pol, and Env (Fig. 2C). The strongest responses, as measured by fraction of IFNγ and TNFα secreting cells following stimulation, were elicited by CD8+ central memory T cells, with most responses targeted against Gag peptides (Fig. 2C, Supplementary Figs. 3, 4). CD8 Tcm cellular responses in the Ad26/MVA and Ad26/MVA + Env groups were statistically significant compared to sham controls (Fig. 2C, *P* = 0.0006 and *P* = 0.0002 for Ad26/MVA and Ad26/MVA + Env groups compared to Sham, respectively, Kruskal–Wallis test with Dunn’s correction for multiple comparisons). Differences between groups for Gag-specific CD4+ and CD8+ T cell populations were not significant (Supplementary Fig. 3). Taken together, these data show that the Ad26/MVA therapeutic vaccine induced CD4+ and CD8+ responses against Gag, Pol, and Env, with particularly strong Gag-specific CD8+ central memory T cells.

To determine whether boosting with SIV Env enhanced Env-specific antibodies elicited by Ad26/MVA therapeutic vaccination, we performed anti-SIV_smE543_ gp140 ELISAs on longitudinal serum samples from each group. Between weeks 4 and 28 post-infection, IgG ELISA titers were comparable in all groups reflective of the brief period of viral replication prior to ART initiation (Fig. 2D). At week 28 (2 weeks after the Ad26 prime) and week 40 (2 weeks following the Ad26 boost) IgG titers in each treatment group were elevated when compared to sham controls (Fig. 2; *P* = 0.0001 and *P* = 0.0005 for the Ad26/MVA and Ad26/MVA + Env groups, respectively, compared to sham for week 28; *P* = 0.0145 and *P* = 0.0052 for the Ad26/MVA and Ad26/MVA + Env groups, respectively, compared to sham for week 40; two-way ANOVA with Bonferroni correction for multiple comparisons). Following the first SIV Env protein boost at week 50, and the second boost at week 60, a significant increase in anti-Env IgG titer was observed between the Ad26/MVA and Ad26/MVA + Env groups at both time points (Fig. 2D, 4.539 and 5.811 log mean anti-Env IgG titers for Ad26/MVA and Ad26/MVA + Env groups, respectively, *P* < 0.0001 at week 50 post-infection, and 4.306 and 5.579 log mean anti-Env IgG titers for Ad26/MVA and Ad26/MVA + Env groups, respectively, *P* ≤ 0.0001 at week 62 post-infection). However, the MVA boost did not substantially increase anti-Env IgG titers (Fig. 2D). Together, these data demonstrate that Ad26/MVA vaccination elicited humoral and cellular immune responses and that the SIV Env boost increased both Env-specific antibody and T cell responses.

### Innate immune stimulation by vesatolimod

**Fig. 3:**
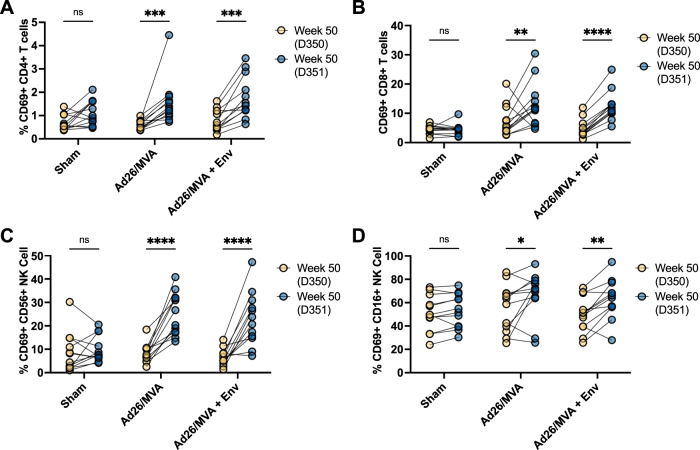
Cellular activation following vesatolimod administration. **A**–**D** T and NK cell activation in both sham and vaccinated groups as measured by increase in cell surface CD69+ expression one day following vesatolimod administration at week 50 post-infection in CD4+ T cells (**A**), CD8+ T cells (**B**), CD56+ NK Cells (**C**), and CD16+ CD56- NK Cells (**D**). Statistical significance determined from two-way ANOVA with Bonferroni correction for multiple comparisons. * < 0.05, ** < 0.01, *** < 0.001, **** < 0.0001.

Vesatolimod was orally administered five times every 2 weeks commencing after the first MVA inoculation with or without the gp140 protein subunit boost in the vaccine groups. Cell-surface CD69 expression on peripheral CD4+ T cell, CD8+ T cell, and multiple NK cell populations increased 24 h following vesatolimod administration (Fig. 3, Supplementary Figs. 5–7), consistent with our prior observations^19,20,23^. Concomitant increases in total serum cytokine concentrations were observed following vesatolimod administration, including Eotaxin, I-TAC, IL-1RA, MCP-1, MIG, and IL-6, in agreement with earlier reports (Supplementary Figs. 8–9)^19,20,23^. Taken together, repeated doses of oral vesatolimod every 2 weeks following the MVA boosts led to activation of CD4+ T cells, CD8+ T cells, and NK cells.

### Viral rebound following ART discontinuation

**Fig. 4:**
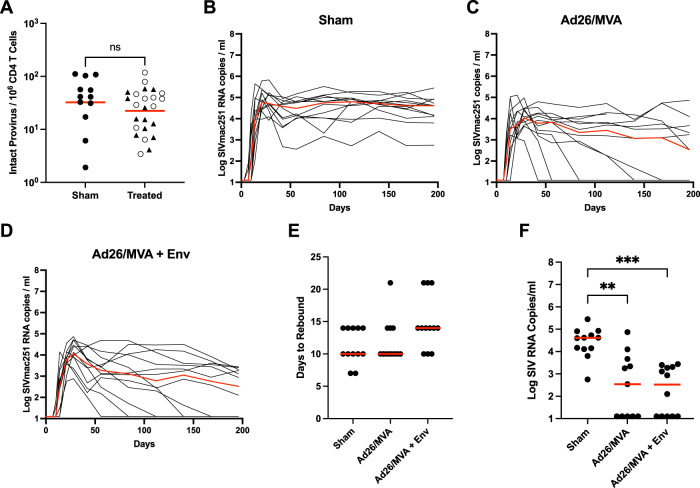
Control of rebound viremia following antiretroviral therapy interruption. **A** Intact proviruses per million CD4+ T cells quantified via an SIV-optimized digital-droplet PCR based Intact Proviral DNA Assay (IPDA) measured at week 72 post-infection, directly before ART interruption. Treatment groups were aggregated into a single group and compared to sham controls. Solid Triangles are animals in the Ad26/MVA treatment group and open circles are animals from the Ad26/MVA + Env treatment group. Red lines show the geometric mean. Statistical significance was derived from a nonparametric Mann–Whitney U Test. **B**–**D** Plasma viral loads (in log SIV_mac251_ *gag* copies/ml plasma) for each study group 198 days following ART interruption at week 72 post-infection. Red curves indicate median values. **E** Number of days until rebound infection was detected after ART interruption at week 72 post-infection. Red lines indicated median values. Statistical significance was determined from a Kruskal–Wallis test with Dunn’s correction for multiple comparisons. **F** Plasma viral loads detected at day 198 post-ART interruption, for each treatment group and sham animals. Red lines denote median values. Statistical significance was determined from a Kruskal–Wallis test with Dunn’s correction for multiple comparisons. * < 0.05, ** < 0.01, *** < 0.001, **** < 0.0001.

At week 72, all animals discontinued ART to determine the therapeutic efficacy of each intervention, and we assessed plasma viral loads for 198 days. Directly prior to ART discontinuation, we also assessed the frequency of intact proviruses in the viral reservoir, reflective of the replication-competent reservoir. Median intact provirus levels in the treated animals trended lower than in sham controls prior to ART discontinuation (Fig. 4A, median 41 vs. 28 intact proviruses per million CD4+ T cells, *P* = 0.2241, Mann–Whitney U test). Intact provirus levels were comparable between virologic controllers and non-controllers on day 198 following ART discontinuation (Supplementary Fig. 10).

All animals demonstrated rapid viral rebound following ART discontinuation. However, 5/12 animals in both the Ad26/MVA and the Ad26/MVA + Env treatment groups exhibited post-rebound virologic control to undetectable levels (Fig. 4B–D). Time to rebound was similar in both vaccine groups and the sham group, consistent with the lack of substantial reduction of intact proviral loads, with a modest delay of a median of 4 days between the Ad26/MVA and the Ad26/MVA + Env groups (Fig. 4E). Ad26/MVA and Ad26/MVA + Env treated animals nevertheless exhibited markedly reduced viral loads on day 198 after ART discontinuation of 2.1 logs (Fig. 4F, median viral loads 4.61, 2.54, and 2.52 log SIV copies/ml for sham, Ad26/MVA, and Ad26/MVA + Env groups, respectively; *P* = 0.0019 sham vs. Ad26/MVA, *P* = 0.0002 sham vs. Ad26/MVA + Env, Kruskal–Wallis test with Dunn’s multiple comparisons). These data demonstrate that Ad26/MVA therapeutic vaccination with or without the SIV Env protein boost led to partial post-rebound virologic control, including control to undetectable levels in 5/12 animals in each intervention group. No appreciable difference in the kinetics of viral rebound or viral control was observed between the two vaccine groups.

### Vaccine-elicited cellular immune responses correlated with virologic control

**Fig. 5:**
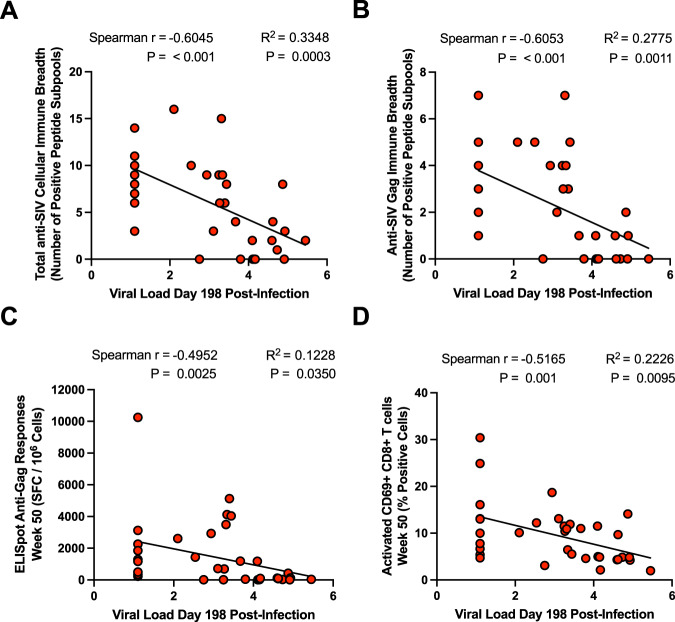
Immune correlates of virologic control following antiretroviral therapy interruption. **A**–**D** Spearman correlation plots between total cellular anti-SIV immune breadth as determined by the number of positive subpools detected via IFNγ ELISpot (**A**), the total anti-Gag Immune breadth as determined by the number of Gag-positive subpools detected via IFNγ ELISpot (**B**), anti-Gag IFNγ ELISpot responses quantified as SFC/10^6^ cells (**C**), frequency of activated (i.e., CD69+ ) anti-viral CD8+ T cells at week 50 post-infection (**D**), and the viral load determined at day 198 after ART interruption. Spearman *r* coefficients and *P* values are displayed above each correlation plot. *R*^2^ values and best fit lines are also displayed following an additional linear regression analysis.

We next performed a correlation analysis to define immunological parameters associated with virologic control at day 198 following ART discontinuation. Multiple cellular immune parameters inversely correlated with setpoint viral load at day 198 post-ART interruption, including total SIV-specific immune breadth, Gag-specific immune breadth, and total Gag-specific cellular responses as measured by ELISpot assays at week 50 (Fig. 5A–C, Supplementary Figs. 11, 12; Spearman *r* = −0.6045, adjusted *P* < 0.001, Spearman *r* = −0.6053, adjusted *P* < 0.001, and Spearman *r* = 0.4952, adjusted *P* = 0.0025, respectively, for correlations described above). The frequency of activated (i.e., CD69+ ) anti-viral CD8+ T cells in the peripheral blood at week 50 also correlated with virologic control (Fig. 5D, Spearman *r* = −0.5165, *P* = 0.001). Gag and Env-specific CD8 Tcm and Tem cell populations at week 62 inversely correlated with viral loads on day 198 post-ART interruption (Supplementary Fig. 11). In contrast, Env-specific antibody titers measured at weeks 50 and 62 post-infection did not significantly correlate with virologic control (Supplementary Fig. 12). Overall, SIV-specific T cell responses and T cell activation were the strongest correlates of virologic control (Supplementary Fig. 12).

## Discussion

In this study, we show that Ad26/MVA therapeutic vaccination with vesatolimod with or without an SIV Env protein boost led to virologic control in ~42% (10/24) of SIV-infected rhesus macaques following ART discontinuation. The addition of the SIV Env protein boost did not appreciably improve therapeutic outcomes, and vaccine-elicited IgG titer did not correlate with virologic control following ART interruption. In contrast, cellular immune responses correlated strongly with virologic control, providing evidence for the importance of T cell immunity for therapeutic vaccine strategies.

Recent work assessing the efficacy of Ad26/MVA therapeutic vaccination in SIV and SHIV infected rhesus macaques has shown promising results^18,24^, although sustained virologic control following ART interruption in humans has not yet been achieved^24^. A recent randomized placebo-controlled double-blinded Phase I Ad26/MVA therapeutic vaccine trial (RV405) reported a modest delay in viral rebound, suggesting that current therapeutic vaccine strategies induce inadequate cellular immune breadth in humans^25^. Additional studies have shown that Ad26 or MVA vaccination in combination with an SIV Env subunit boost induced robust cellular immune responses in uninfected rhesus macaques, proving a means to improve overall cellular immune breadth^26,27^. Furthermore, a recent trial consisting of HIV-1 controllers given a total of ten doses of vesatolimod while virologically suppressed demonstrated a modest delay in viral rebound following ART interruption of approximately one week with four individuals from the treatment group showing a delay in rebound of more than 6 weeks^22^. We have also recently reported that administration of the monoclonal antibody PGT121 during ART suppression with vesatolimod delayed viral rebound^23^.

Animals receiving the SIV Env protein boost exhibited higher Env-specific antibody responses compared with Ad26/MVA vaccination with vesatolimod alone, but did not induce neutralizing antibodies against the neutralization-resistant SIVmac251 challenge virus. We did not observe additional therapeutic efficacy with the addition of the Env boost, suggesting that binding antibody titers were not the primary mechanism of post-rebound virologic control. The strongest correlates of virologic control were cellular immune breadth and activated CD8+ T cells, consistent with results with a different therapeutic vaccine^28^.

Inducing bNAb via vaccination is a critical objective in HIV vaccine research^29^ and may require complex regimens^30–36^. Fc effector functional antibodies also contribute to HIV-1 and SIV immunity^37–39^. In our study, we did not observe an improvement in therapeutic efficacy in animals that received the Env gp140 boost, but future studies could evaluate the contributions of Fc effector functions associated with antibodies elicited by therapeutic vaccination strategies.

The size of the viral reservoir as measured by the SIV Intact Provirus DNA Assay (IPDA) in CD4+ T cells was comparable in vaccinated animals and sham animals prior to ART interruption. These data suggest that therapeutic vaccination with TLR7 activation did not directly target the viral reservoir, but rather functioned by enhancing cellular immunity leading to post-rebound virologic control. In humans, however, vesatolimod treated HIV-1 controllers on suppressive ART exhibited a small but significant reduction in replication-competent provirus and a modest delay in the time to viral rebound^22^.

In summary, our data demonstrate that Ad26/MVA therapeutic vaccination with or without an SIV Env boost with vesatolimod administration led to robust SIV-specific cellular immune responses and post-rebound virologic control following ART discontinuation. These results provide a rationale for further studies involving Ad26/MVA therapeutic vaccine regimens for an HIV-1 functional cure.

## Materials and methods

### Animals

36 outbred young adult male and female rhesus macaques of Indian origin were selected for this study. All animals were screened for expression of protective MHC class I alleles Mamu-A*01, Mamu-B*08, and Mamu-B*17, and animals with a positive genotype were removed from the study. Animals were evenly distributed across groups based on age, gender, and protective or susceptible *TRIM5α* alleles. Animals were housed and cared for by veterinary professional at Bioqual in Rockville, Maryland. Animals were intrarectally infected with 500 TCID50 of SIV_mac251_ challenge stock, as in previous reports^18^. All animal assays were performed blinded, and procedures were overseen and approved by the Bioqual Institutional Animal Care and Use Committee.

### ART regimen

Antiretroviral therapy (ART) for this study comprised of a preformulated cocktail of 5.1 mg/ml TDF, 40 mg/ml emtricitabine (FTC), and 2.5 mg/ml DTG dissolved with 15% (v/v) kleptose at pH 4.2. ART cocktail was administered once daily at 1 ml/kg body weight via the subcutaneous route.

### Therapeutic Ad26/MVA and Ad26/MVA + Env vaccination

In Ad26/MVA and Ad26/MVA + Env groups, animals were inoculated through the intramuscular (IM) route with 3 × 10^10^ viral particles of Ad26 vectors expressing SIV_smE543_ *gag–pol–env* immunogens at weeks 24 and 36 and with 10^8^ plaque-forming units of MVA vectors expressing the same SIVs_mE543_ *gag–pol–env* immunogens at weeks 48 and 60. Ad26/MVA + Env animals were inoculated with 250 μg SIV_mac251_ gp140 with aluminum phosphate adjuvant at weeks 48 and 60 with the MVA boosts. Sham animals were given saline IM. In the Ad26/MVA and Ad26/MVA + Env treatment groups, animals received 10 administrations of 0.1 mg/kg vesatolimod (GS-9620, Gilead Sciences) by oral gavage every 2 weeks from weeks 50–58 to 62–70.

### qRT-PCR viral load assay

Viral RNA was isolated using a QIAcube HT and the cador Pathogen 96 QIAcube HT Kit (QIAGEN, Germany). RNA standards were generated using the Simian immunodeficiency virus (SIV) gag gene sequence as template and in vitro transcribed with the AmpliCap-Max T7 High Yield Message Maker Kit (Cell Script). Standard RNA was purified using the RNA clean and concentrator kit (Zymo Research). Log dilutions of the standard were prepared and run for each RT-PCR assay. Reverse transcription of standards and samples was performed using Superscript VILO (Invitrogen). The following primers were used for RT-PCR: forward primer 5′-GTCTGCGTCATCTGGTGCATTC-3′, reverse primer 5′-CACTAGGTGTCTCTGCACTATCTGTTTTG-3′, and fluorescently labeled probe 5′-CTTCCTCAGTGTGTTTCACTTTCTCTTCTGCG-3′. Samples and standards were run in duplicate on a Quantstudio 6 Flex system (Applied Biosystems) using the following thermocycle settings: 95 °C for 20 s for initial denaturation, then 95 °C for 1 s followed by 60 °C for 20 s repeated for 45 cycles. Viral loads were calculated as RNA copies per ml and the assay sensitivity was 250 copies/ml.

### IFNγ enzyme-linked immunospot (ELISPOT) assay

ELISPOT plates were coated with mouse anti-human IFNγ monoclonal antibody from BD Pharmigen at 5 µg/well and incubated overnight at 4 °C. Plates were washed with DPBS wash buffer (DPBS with 0.25% Tween20), and blocked with R10 media (RPMI with 10% heat inactivated FBS with 1% of 100× penicillin-streptomycin) for 1–4 h at 37 °C. SIVmac239 peptides (JPT) were prepared & plated at a concentration of 1 µg/well, and 200,000 cells/well were added to the plate. The peptides and cells were incubated for 18–24 h at 37 °C. All steps following this incubation were performed at room temperature. The plates were washed with ELISpot wash buffer (11% 10× DPBS and 0.3% Tween20 in 1 L MilliQ water) and incubated for 2 h with Rabbit polyclonal anti-human IFN-γ Biotin from U-Cytech (1 µg/mL). The plates were washed a second time and incubated for 2 h with Streptavidin-alkaline phosphatase from Southern Biotech (2 µg/mL). The final wash was followed by the addition of Nitor-blue Tetrazolium Chloride/5-bromo-4-chloro 3’indolyphosphate p-toludine salt (NBT/BCIP chromagen) substrate solution for 7 min. The chromagen was discarded and the plates were washed with water and dried in a dim place for 24 h. Plates were scanned and counted on a Cellular Technologies Limited Immunospot Analyzer.

### Intracellular cytokine staining (ICS) assay

10^6^ PBMCs/well were re-suspended in 100 µL of R10 media supplemented with CD49d monoclonal antibody (1 µg/mL). Each sample was assessed with mock (100 µL of R10 plus 0.5% DMSO; background control), SIVmac239 peptides (2 µg/mL), &/or 10 pg/mL phorbol myristate acetate and 1 µg/mL ionomycin (Sigma-Aldrich) (100 µL; positive control) and incubated at 37 °C for 1 h. After incubation, 0.25 µL of GolgiStop and 0.25 µL of GolgiPlug in 50 µL of R10 was added to each well and incubated at 37 °C for 8 h and then held at 4 °C overnight. The next day, the cells were washed twice with 2% FBS/DPBS buffer and stained with predetermined titers of mAbs against CD95 (clone DX2, PE), CD28 (clone L293, PERCP.Cy5.5), CD4 (clone L200, AMCYAN), CD8 (clone SK1, APC Cy7) for 30 min. Cells were then washed twice with 2% FBS/DPBS buffer and incubated for 15 min with 200 µL of BD CytoFix/CytoPerm Fixation/Permeabilization solution. Cells were washed twice with 1X Perm Wash buffer (BD Perm/Wash^TM^ Buffer 10X in the CytoFix/CytoPerm Fixation/ Permeabilization kit diluted with MilliQ water and passed through 0.22 µm filter) and stained with intracellularly with mAbs against TNFα (clone Mab11, FITC), CD69 (clone TP1.55.3, ECD), IFNγ (clone B27, PE-Cy7), IL2 (clone MQ1-17H12, APC), CD3 (clone SP34.2, A700), for 30 min. Cells were washed twice with 1X Perm Wash buffer and fixed with 250 µL of freshly prepared 1.5% formaldehyde. Fixed cells were transferred to 96-well round bottom plate and analyzed by BD FACSymphony^TM^ system.

### SIV gp140 IgG ELISA

Ninety-six-well Maxisorp ELISA plates (Thermo Fisher Scientific) were coated overnight with 100 μl/well of 1 μg/mL SIVmac32H gp140 protein in phosphate-buffered saline (Gibco), washed and then blocked for 2 h with blocker casein in PBS (Thermo Scientific). Macaque sera were then added in threefold serial dilutions and incubated for 1 h at room temperature. The plates were washed three times with PBS containing 0.05% Tween 20 and incubated for 1 h with a 1/1000 dilution of a horseradish peroxidase-conjugated goat anti-human secondary antibody (Jackson Immunoresearch labs). The plates were washed three times and developed with SureBlue tetramethylbenzidine microwell peroxidase (KPL Research Products), stopped by the addition of stop solution (KPL Research products), and analyzed at 450 nm with a Versamax ELISA microplate reader (Molecular Devices) using Softmax Pro 6.5.1 software. ELISA titers were defined as the highest reciprocal serum dilution that yielded an OD450nm absorbance >0.2.

### Intact proviral DNA assay

CD4+ T cells were isolated from viably frozen peripheral mononuclear cells (PBMCs) using the EasySep Non-Human Primate CD4+ T Cell Isolation Kit (Stem Cell Technologies) according to the manufacturer’s protocol. Total genomic DNA was extracted from the isolated T cells using a QIAamp DNA mini kit (Qiagen), according to manufacturer’s protocol. DNA concentration was measured using a Nanodrop spectrophotometer (Thermo Fisher Scientific). The SIV IPDA, consisting of three separate multiplex PCR reactions, was performed on a Bio-Rad droplet digital PCR system (ddPCR)^40,41^. The first ddPCR reaction uses a duplex primer/probe mix to specifically quantify intact SIV genomes by targeting two amplicons located in *pol* and *env*. The reaction also uses a second set of unlabeled competition probes to exclude defective proviruses that are hypermutated at key positions. The second ddPCR reaction quantifies unintegrated 2-LTR circles by multiplexing primers and probes that target a region unique to the 2LTR junction^42^ with the IPDA *env* amplicon. The third ddPCR reaction targets two amplicons located within the housekeeping gene *RPP30* in order to quantify input cell numbers. The amplicons are spaced the same distance apart as the IPDA amplicons and are also used to quantify and correct for DNA shearing using the ratio of *single-positive to d*ouble-positive events.

DNA input for the first two reactions was standardized to 55 ng/μL when possible, and a maximum of 302.5 ng DNA was added to each reaction. For the *RPP30* reaction, a total of 3 ng of input DNA was assayed per reaction. All reactions were made with 2X Bio-Rad ddPCR Supermix for Probes in a total volume of 20 uL. Reactions were set up in duplicate or triplicate depending on the amount of available sample DNA. Droplets were generated using the QX200 AutoDG (Bio-Rad) and then subjected to the following cycling conditions: 10 min at 95 °C, 50 cycles of 30 s at 94 °C and 2 min at 56 °C, 10 min at 98 °C, and a final hold at 4 °C. Data analysis was performed using QuantaSoft Analysis Pro software (Bio-Rad). After correcting for DNA shearing and subtracting 2LTR-*env* double-positive events, the final data are reported as frequency of intact proviruses per million CD4+ T cells.

### Plasma cytokine analysis

EDTA plasma cytokine levels were determined using the ProcartaPlex multiplex immunoassay (Thermo Fisher) according to the manufacturer’s instructions, for 15 cytokines: IFN-a, IFN-g, IL-1B, IL-1RA, IL-12p40, IL-2, IL-6, IL-8, IL-10, I-TAC, MCP-1, MIG, and TNF-a. Samples were then read on a Luminex 200 platform and analyzed using Bio-plex Manager software (Bio-Rad).

### Correlational analysis

Pairwise spearman rank coefficients (r) were calculated using the psych package vr. 2.0.12 (https://cran.r-project.org/web/packages/psych/index.html) in R using the corr.test function with default settings. The *adjust* argument was set to “fdr” to calculate adjusted *p* values using Benjamini-Hochberg correction for multiple comparisons. The resulting correlation matrix was visualized as a correlogram using the corrplot package in R (https://cran.r-project.org/web/packages/corrplot/index.html). Spearman rank coefficients were ordered via hierarchical clustering by setting the *order* argument to “hclust” in the corrplot function.

### Statistical analysis

Virological and immunological data analysis and statistical inference testing was performed using GraphPad Prism Version 9.2.0 (GraphPad Software). Correlational analysis was performed in RStudio running R Version 4.0.4. Sample sizes were not predetermined by prior power analysis. Investigators were not blinded to the allocation of samples during the experiments and outcome assessment.

## Supplementary information

Supplement
