# Supplementary material for: Therapeutic efficacy of an Ad26/MVA vaccine with SIV gp140 protein and vesatolimod in ART-suppressed rhesus macaques
Source: NPJ Vaccines. 2022 May 18;7:53. doi: 10.1038/s41541-022-00477-x (PMC9117189; doi:10.1038/s41541-022-00477-x)
Supplement: Supplementary file 1 — Supplement [file 41541_2022_477_MOESM1_ESM.pdf]

Supplementary Information

Therapeutic efficacy of an Ad26/MVA vaccine with SIV gp140 protein and vesatolimod in ART-suppressed rhesus macaques

John D. Ventura[1], Joseph P. Nkolola[1], Abishek Chandrashekar[1], Erica N. Borducchi[1], Jinyan Liu[1], Noe B. Mercado[1], David L. Hope[1], Victoria M. Giffin[1], Katherine McMahan[1], Romas Geleziunas[2], Jeffrey P. Murry[2], Yunling Yang[2], Mark G. Lewis[3], Maria G. Pau[4], Frank Wegmann[4], Hanneke Schuitemaker[4], Emily J. Fray[5], Mithra R. Kumar[5], Janet D. Siliciano[5], Robert F. Siliciano[5], Merlin L. Robb[6], Nelson L. Michael[6], and Dan H. Barouch[1,7†]

[1] Center for Virology and Vaccine Research, Beth Israel Deaconess Medical Center, Harvard Medical School, Boston, MA 02215, USA
[2] Gilead Sciences, Foster City, California 94404, USA
[3] Bioqual, Rockville, Maryland 20852, USA
[4] Janssen Infectious Diseases and Vaccines, 2301 Leiden, The Netherlands
[5] Department of Medicine, Johns Hopkins University School of Medicine, Baltimore, MD, USA
[6] US Military HIV Research Program, Walter Reed Army Institute of Research, Silver Spring, Maryland 20910, USA
[7] Ragon Institute of MGH, MIT, and Harvard, Cambridge, Massachusetts 02139, USA

[†]Correspondence: Dan H. Barouch (dbarouch@bidmc.harvard.edu)

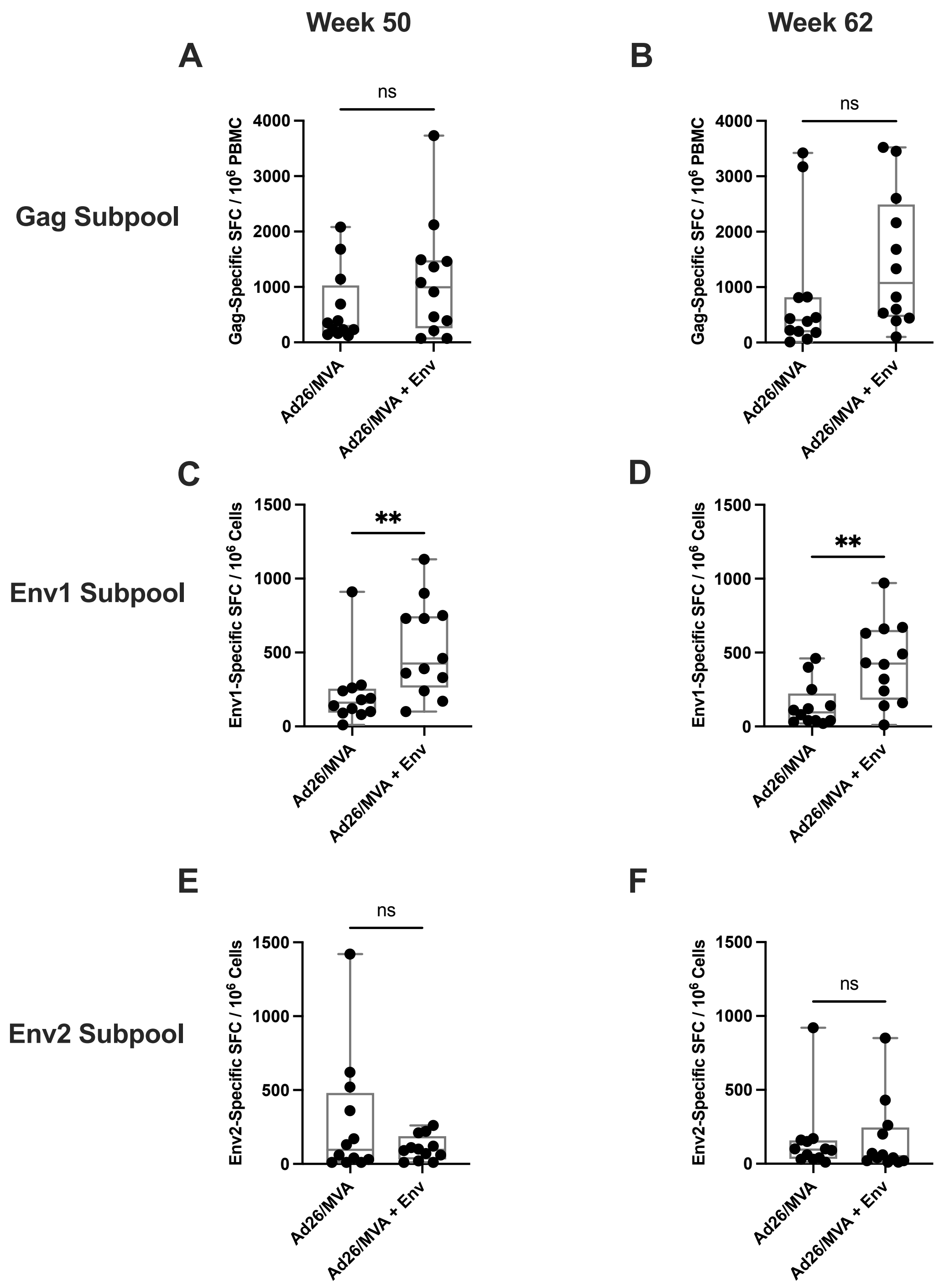

Supplementary Figure 1: SIV Gag-specific and Env-specific cellular responses at weeks 50 and 62 post-infection. SIV Gag-specific cellular immune responses at week 50 (A) and 62 (B) post-infection and SIV Env-specific cellular immune responses at week 50 (C,E) and 62 (D,F) post-infection as measured by IFNγ ELISpot assays following stimulation of PBMCs with the Gag, Env1, and Env2 peptide subpools. Statistical significance determined by Mann-Whitney U test. Red lines indicate median values. SFC (spot forming cells).

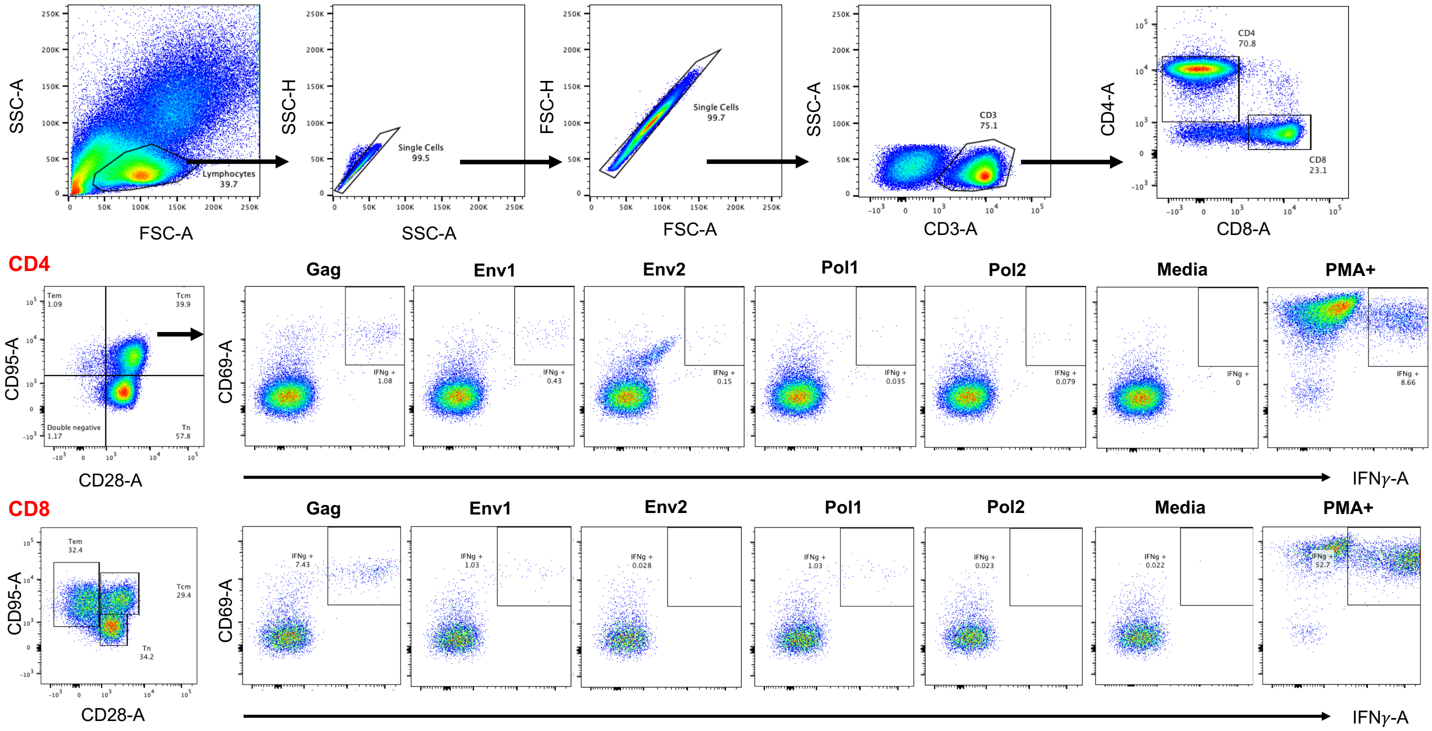

Supplementary Figure 2: Intracellular cytokine staining gating strategy. Representative gating strategy for the multiparameter flow cytometric intracellular cytokine secretion (ICS) assay used in this study.

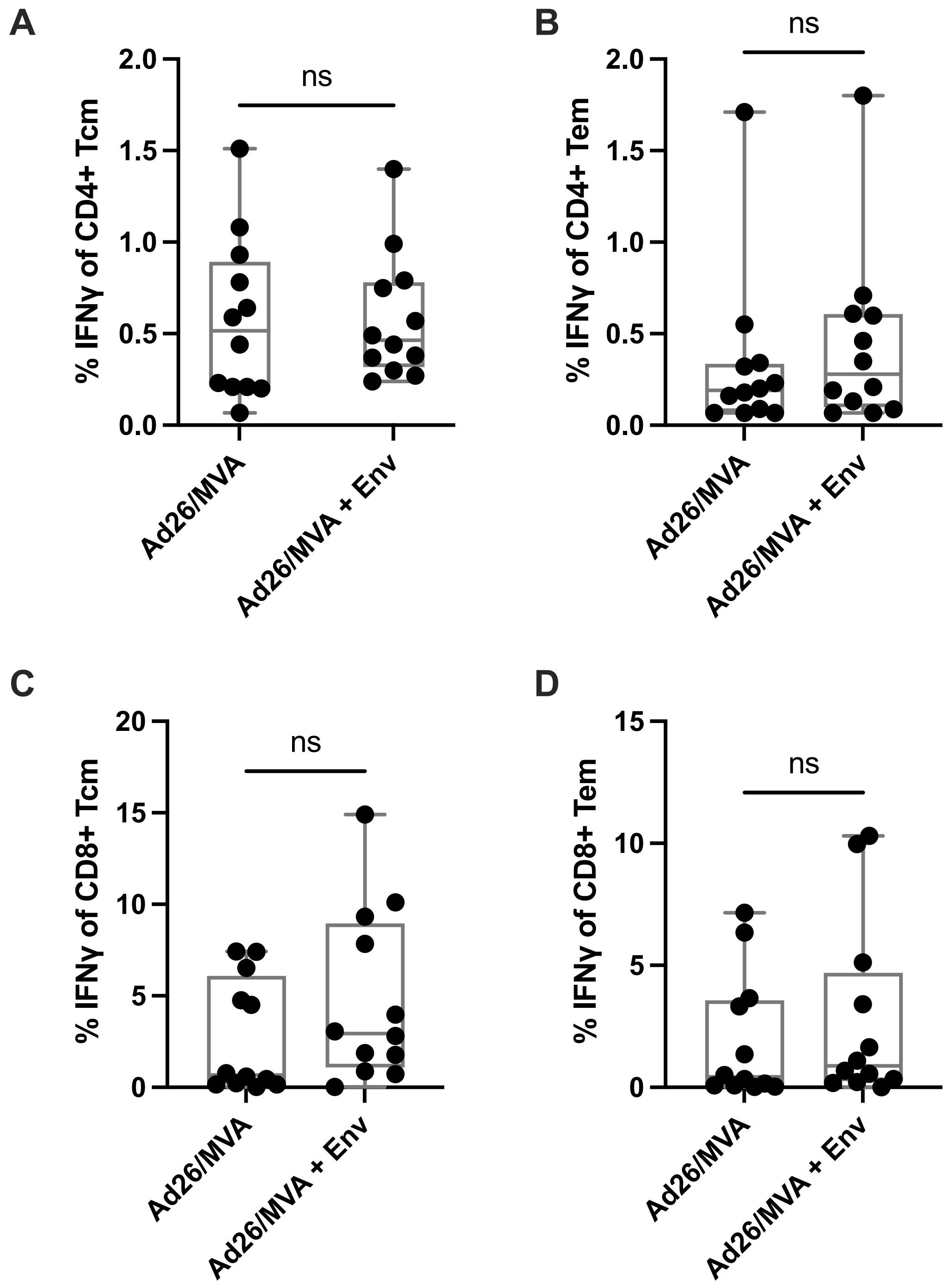

Supplementary Figure 3: Gag-specific cellular responses between the Ad26/MVA and Ad26/MVA + Env groups assessed by intracellular cytokine staining. (A-D) Gag-specific IFNγ-secreting CD4+ Tcm (A), CD4+ Tem (B), CD8+ Tcm (C), and CD8+ Tem (D) populations from PBMC sampled at week 62 post-infection in the Ad26/MVA and Ad26/MVA + Env treatment groups. Statistical significance determined from Mann-Whitney U tests. * < 0.05, ** < 0.01, *** < 0.001, **** <0.0001.

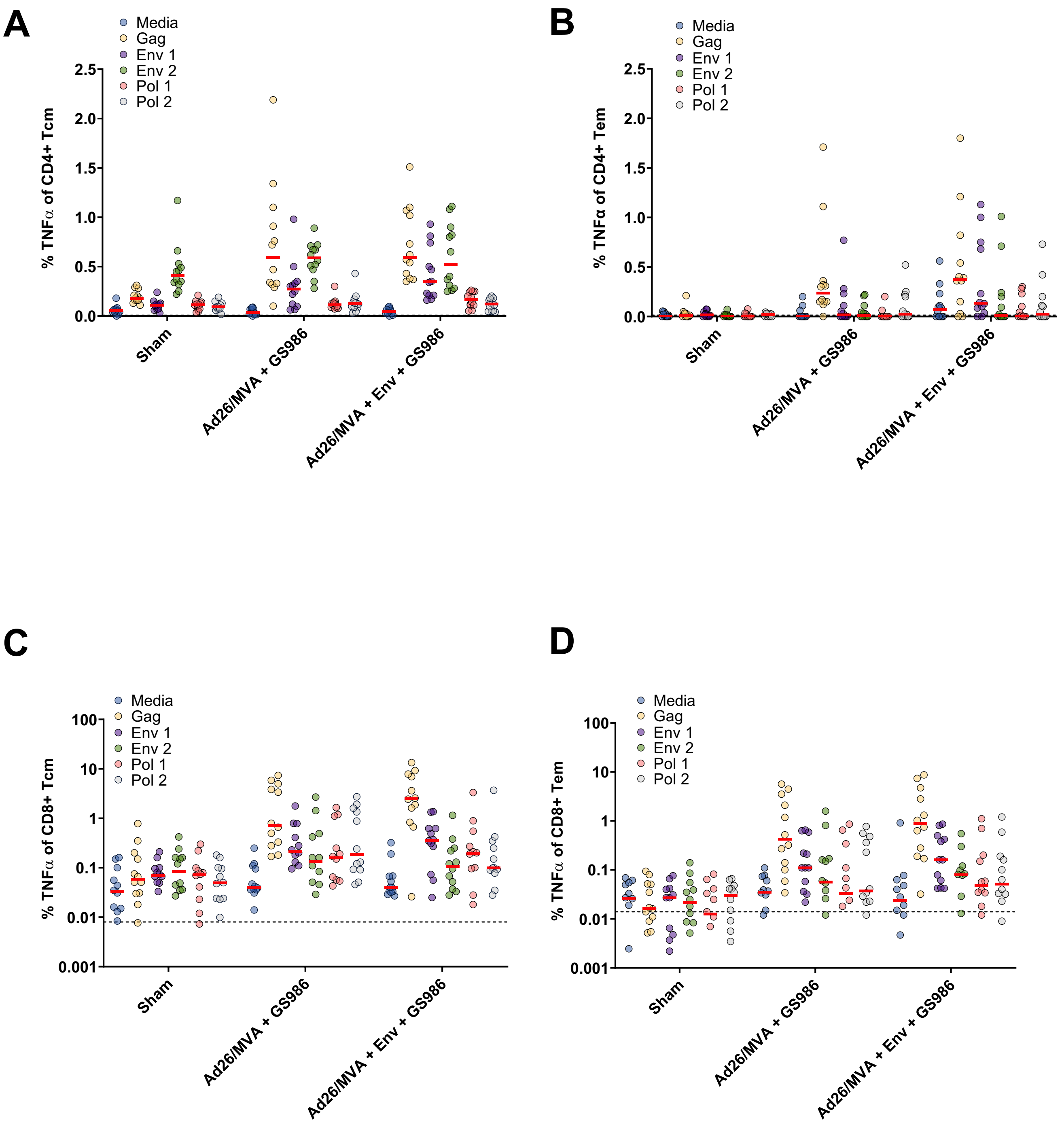

Supplementary Figure 4: SIV-specific TNFα secretion following stimulation with SIV_{mac251} antigens. (A-B) SIV-specific TNFα secretion in CD4+ central (A) and effector (B) memory T cell subsets across sham and all treatment groups. (C-D) SIV-specific TNFα secretion in CD8+ central (C) and effector (D) memory T cell subsets. Measurements were taken from peripheral blood mononuclear cells (PBMCs) sampled at week 62 post-infection. Red lines indicate median values.

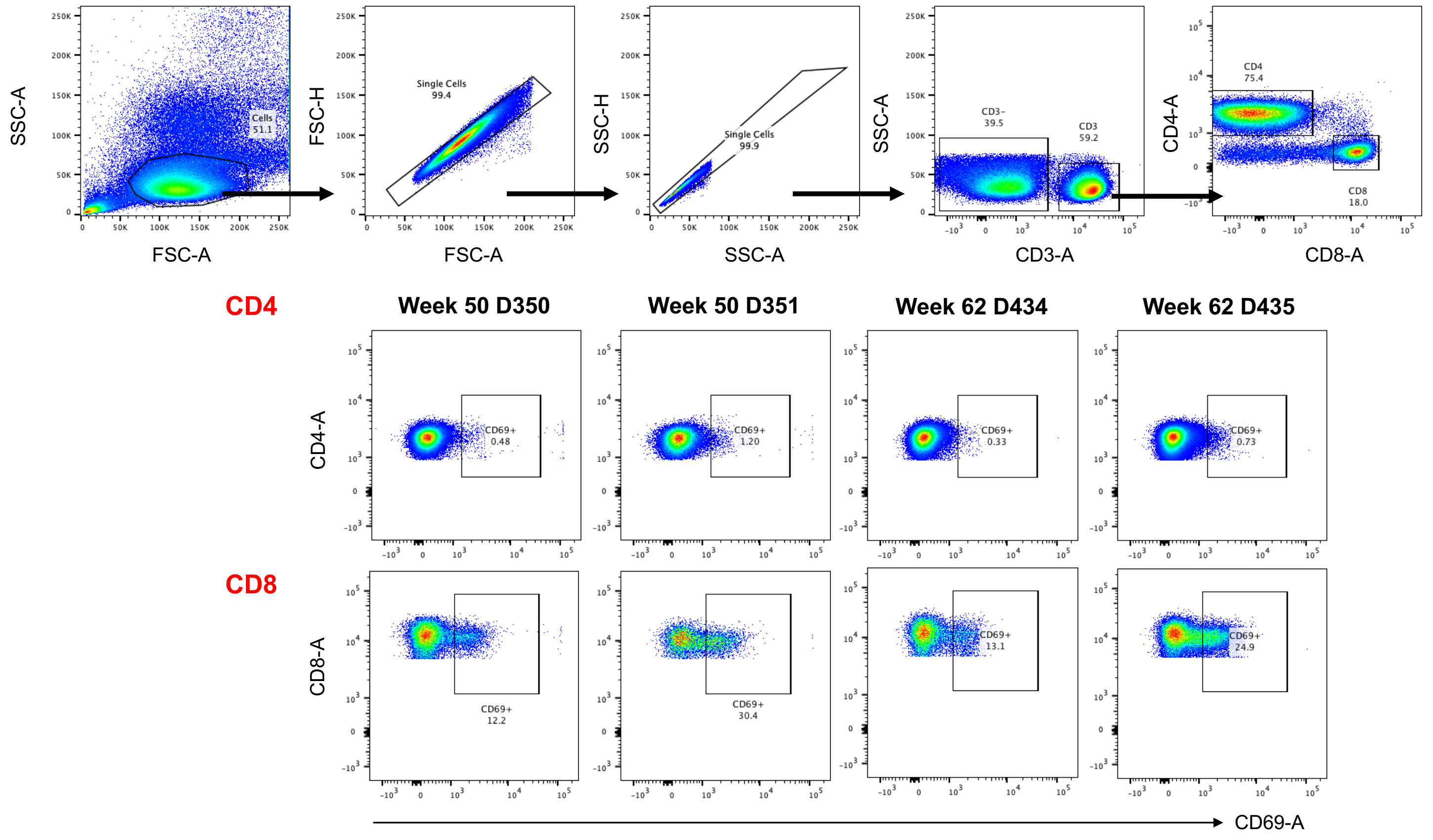

Supplementary Figure 5: Flow cytometric gating strategy to assess T cell activation following vesatolimod administration. Representative gating strategy for determining T cell activation via CD69 surface expression at weeks 50 and 62 post-infection.

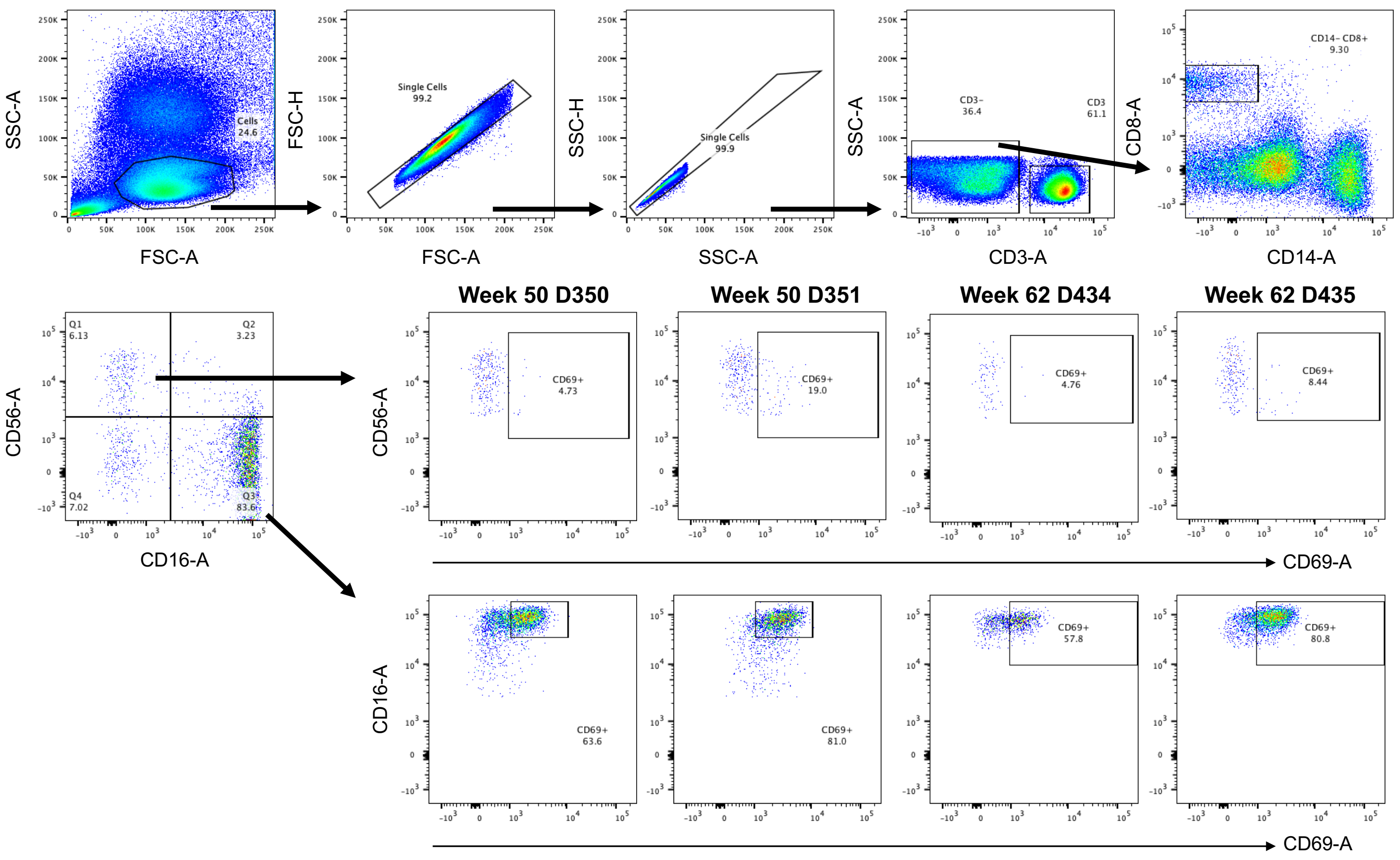

Supplementary Figure 6: Flow cytometric gating strategy to assess NK cell activation following vesatolimod administration. Representative gating strategy for determining NK cell activation via CD69 surface expression of CD69 in CD56+ and CD16+ subsets at weeks 50 and 62 post-infection.

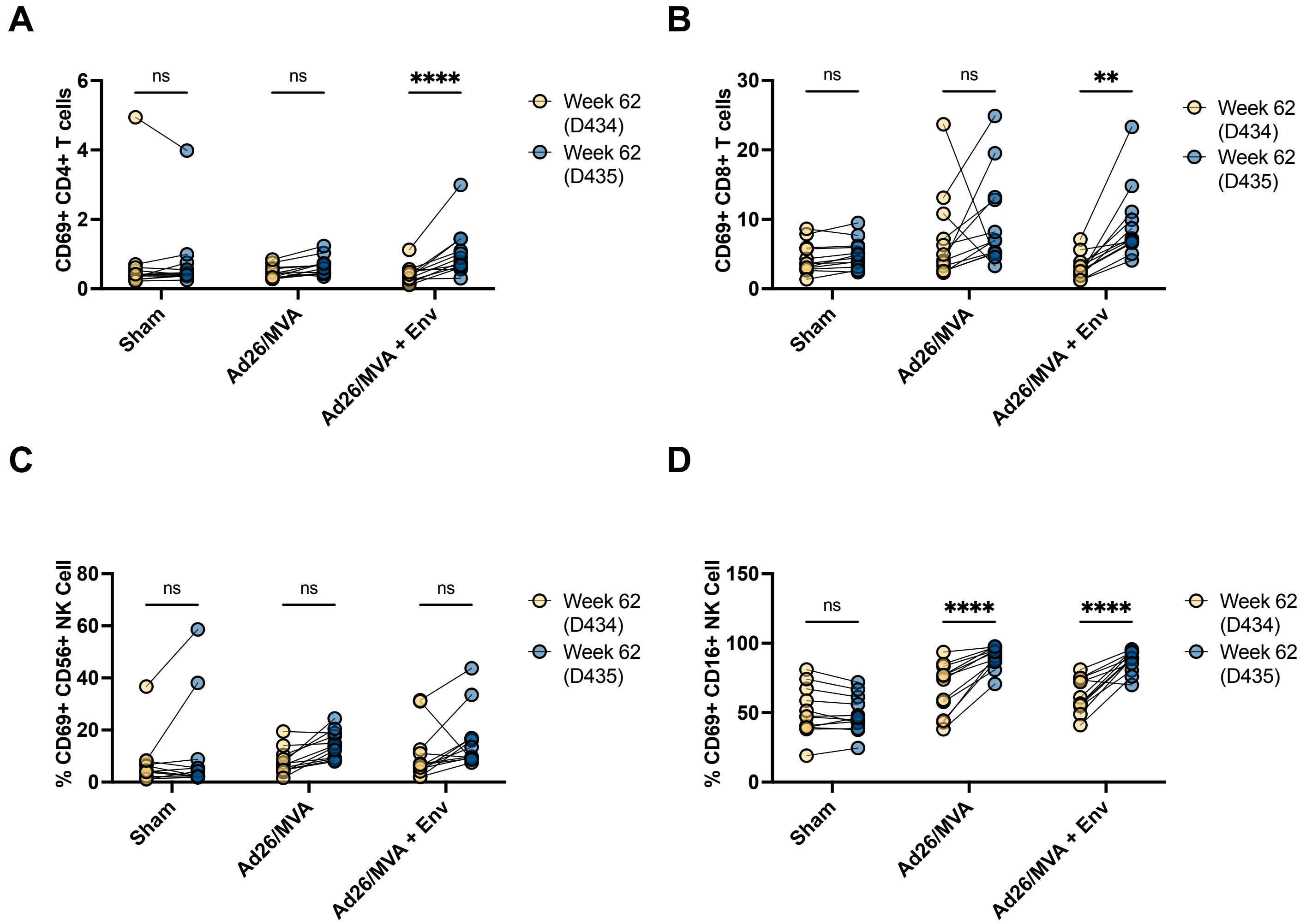

Supplementary Figure 7: Cellular activation following vesatolimod administration at Week 62. (A-H) T and NK cell activation in both sham and vaccinated groups as measured by increase in cell surface CD69+ expression one day following vesatolimod administration at week 62 post-infection in CD4+ T cells (A), CD8+ T cells (B), CD56+ NK Cells (C), and CD16+ CD56- NK Cells (D). Statistical significance determined from two-way ANOVA with Bonferroni correction for multiple comparisons. ∗ < 0.05, ∗∗ < 0.01, ∗∗∗ < 0.001, ∗∗∗∗ <0.0001. Red lines indicate median values.

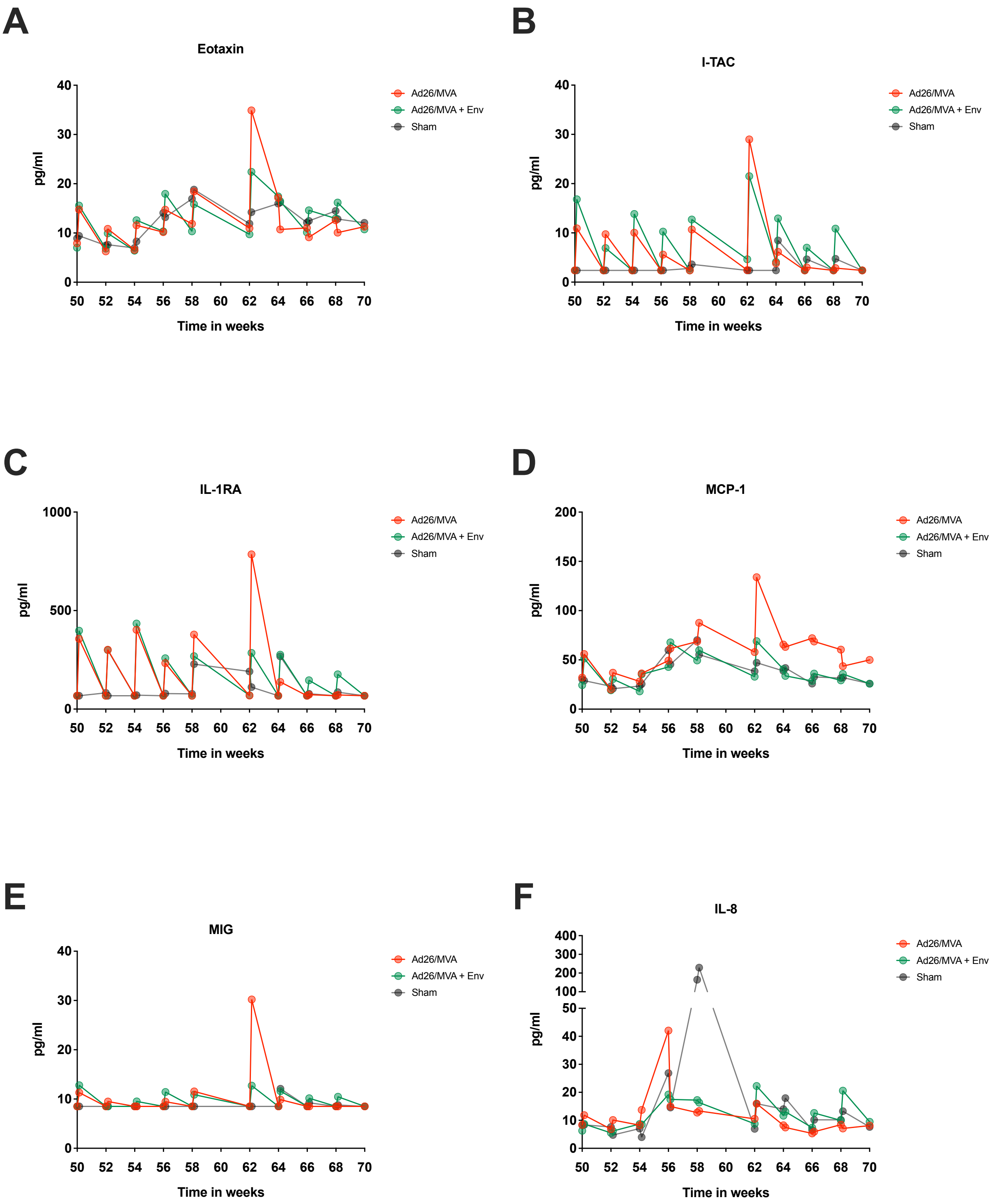

Supplementary Figure 8: Elevation of serum cytokine levels following vesatolimod administration. (A-F) Longitudinal serum cytokine levels (in pg/ml sera) in animals from all study groups from all vesatolimod administrations across the entire ten dose regimen (weeks 50-70).

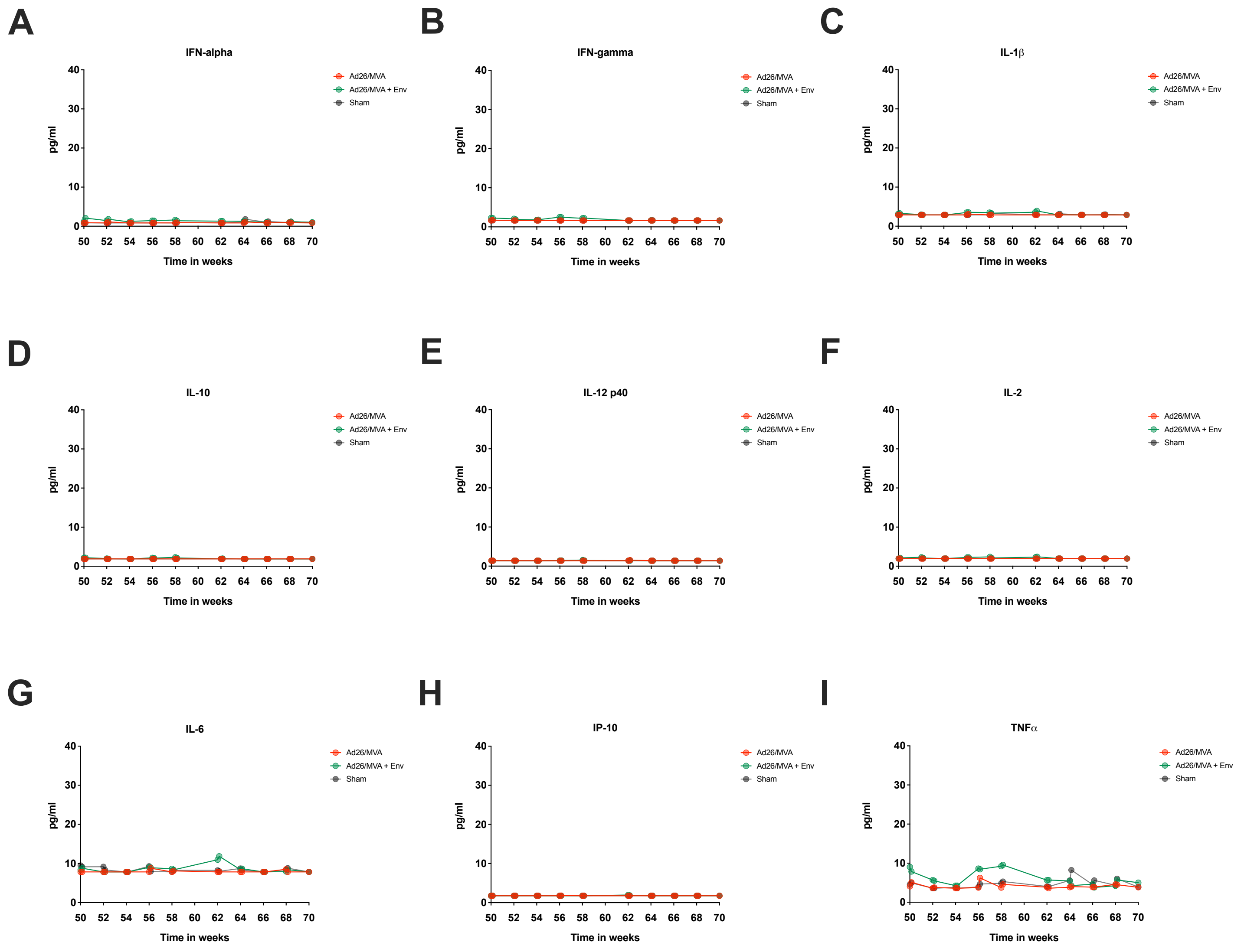

Supplementary Figure 9: Non-elevated serum cytokine levels following vesatolimod administration. (A-I) Longitudinal serum cytokine levels (in pg/ml sera) in animals from all study groups from all vesatolimod administrations that showed no change in serum concentration after the full ten dose regimen (weeks 50-70).

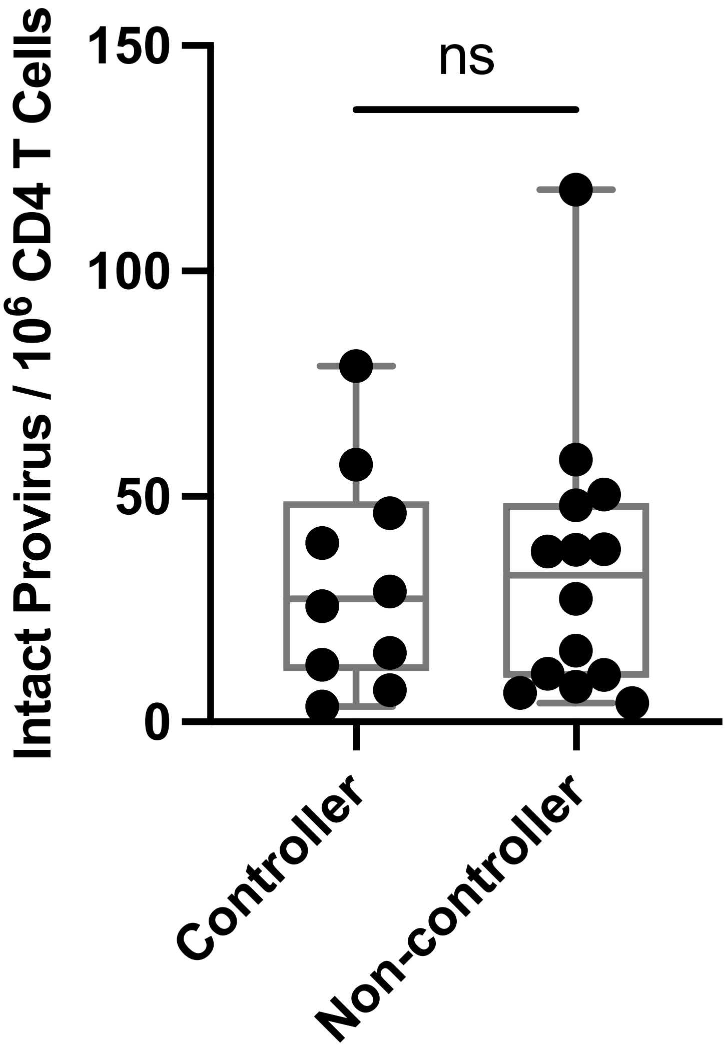

Supplementary Figure 10: Intact reservoir in virologic controllers versus non-controllers. Intact proviruses per million cells measured in CD4+ T cells enriched from PBMC sampled directly prior to ART interruption (ATI), i.e. week 72 post-infection, in animals exhibiting virologic control after 198 days post- ATI versus non-controllers. Intact proviruses were measured by the SIV IPDA. Data shown as a box and whisker plot displaying the max and min values, median, and quartiles. Statistical significance determined by a non-parametric Mann-Whitney U Test.

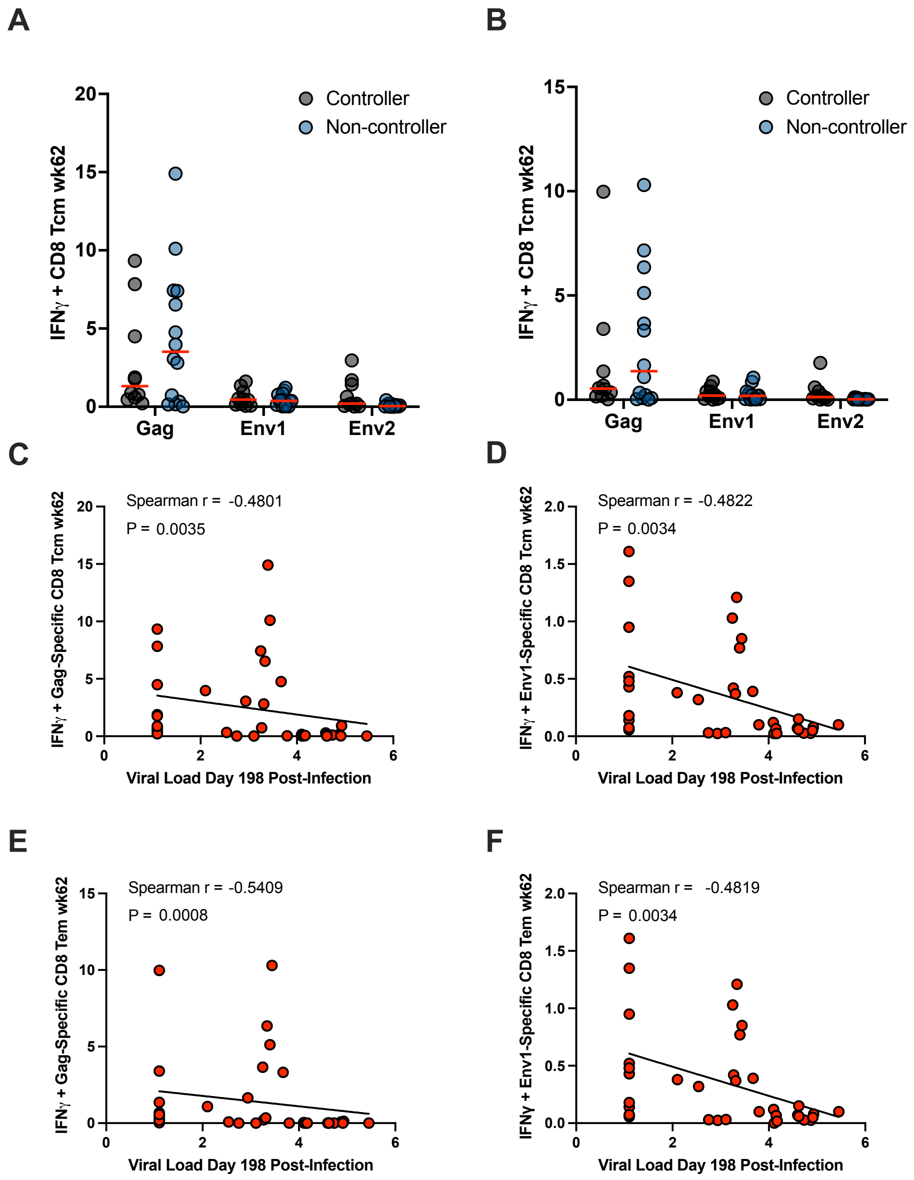

Supplementary Figure 11: Gag/Env Specific CD8 T cell immune responses and CD8+ T cell correlates of virologic control. (A-B) Gag-specific (A) and Env-specific (B) responses measured by intracellular cytokine staining as IFNγ-secreting CD8+ T cells following peptide stimulation in virologic controllers versus non-controllers. (C-F) Statistically significant Spearman correlations between IFNγ-secreting CD8+ central memory Gag and Env1-specific T cells (C-D) and IFNγ-secreting CD8+ effector memory Gag and Env1-specific T cells (E-F) and viral load at Day 198 post-ART interruption. Spearman r and P value are shown above all correlation plots and all correlation plots show a best fit line. Red lines denote median values.

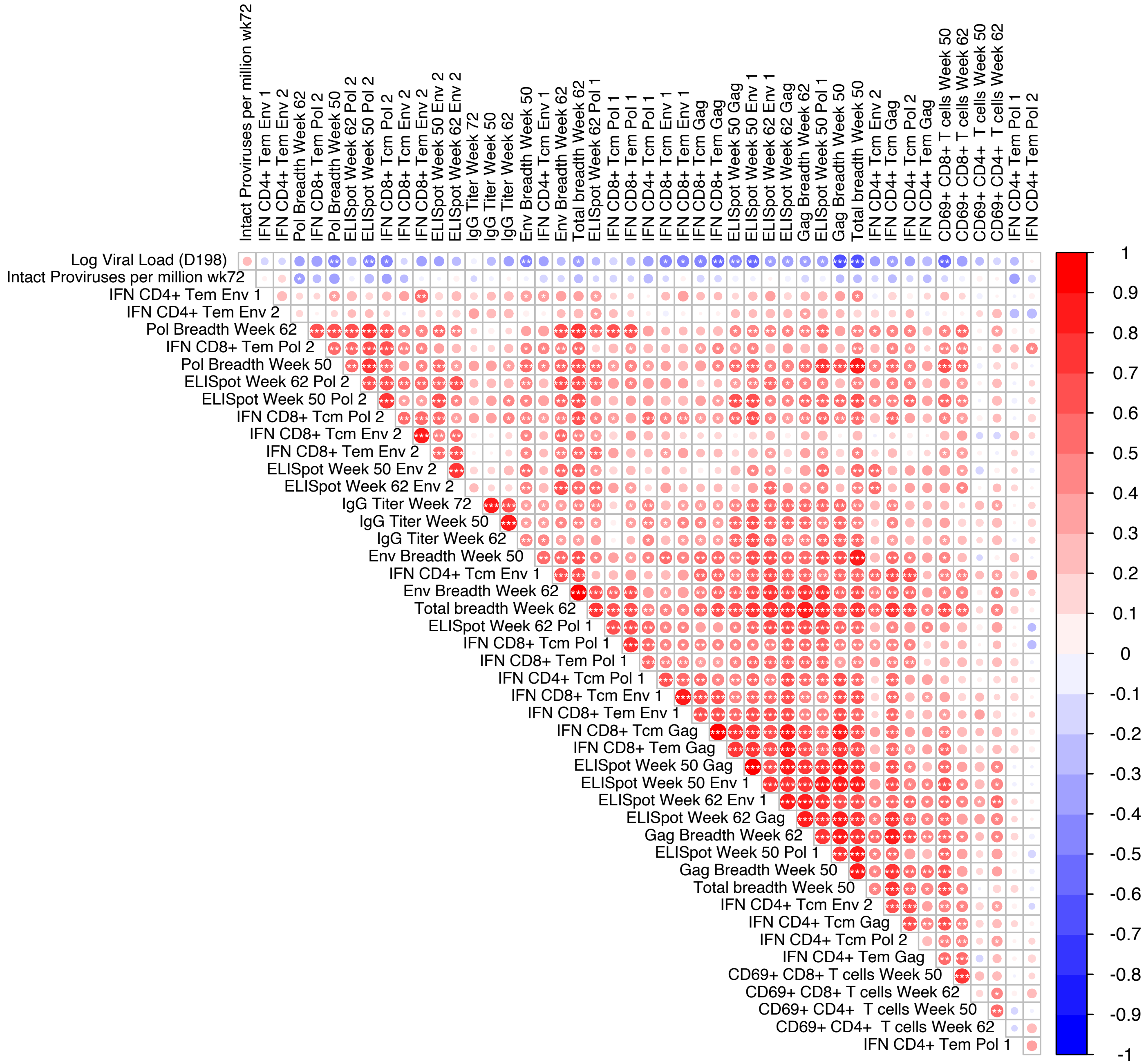

Supplementary Figure 12: Correlative analysis of immune correlates of virologic control following antiretroviral therapy interruption. Correlogram displaying Spearman correlations calculated between the immune parameters measured across the study. Positive and negative correlations are indicated via red and blue circles, respectively, with the area of the circle and shade of the color corresponding to the strength of the correlation. Stars are displayed on statistically significant correlations with p values adjusted via Benjamini-Hochberg false discovery rate correction. $* < 0.05$, $** < 0.01$, $*** < 0.001$.
